# Supplementary material for: R-loop Mapping and Characterization During Drosophila Embryogenesis Reveals Developmental Plasticity in R-loop Signatures
Source: J Mol Biol. Author manuscript; Available in PMC 2022 Jul 15. (PMC9254486; doi:10.1016/j.jmb.2022.167645)
Supplement: Supplemental material [file NIHMS1818083-supplement-Supplemental_material.pdf]

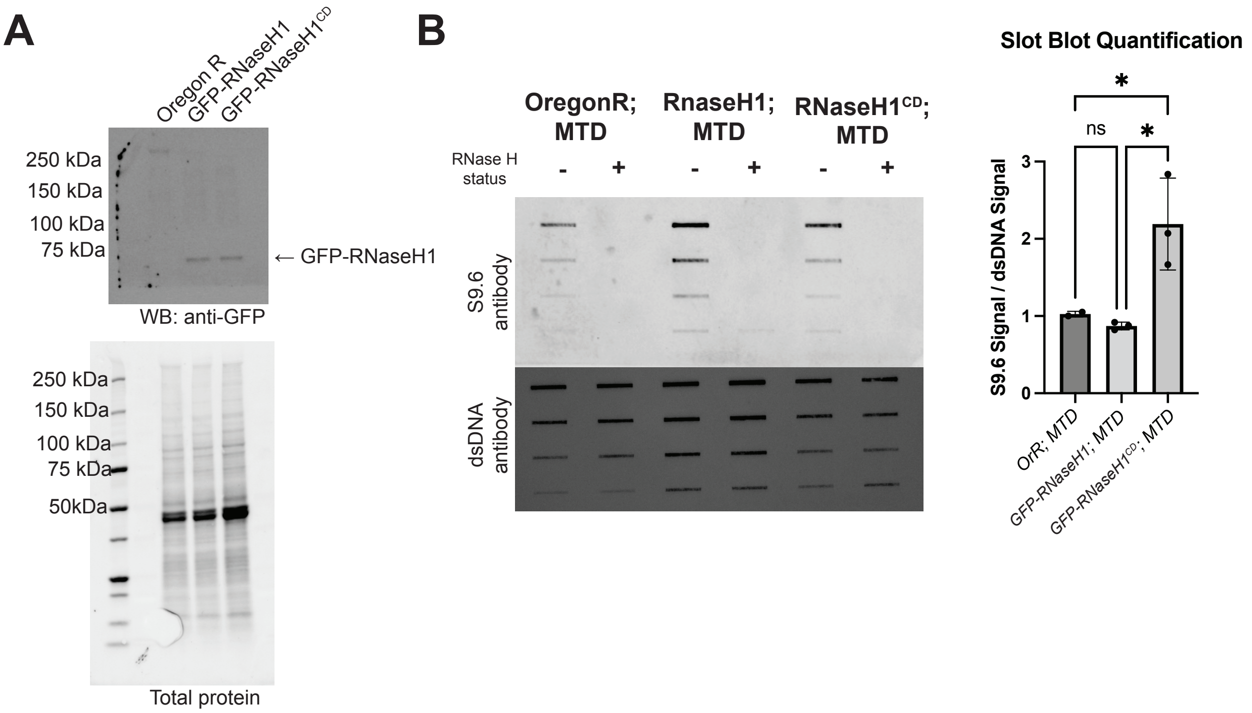

Supplemental Figure 1: Expression of RNaseH1 constructs in early embryos. (A) Western blot (anti-GFP) showing maternally deposited GFP-RNaseH1 and GFP-RNaseH1^{CD} in 0-6 hour embryos. Expected size of GFP + RNaseH1 = 65.1 kDa. (B) Representative slot blot of RNA:DNA hybrid levels, measured by S9.6 antibody intensity, from 2-6h embryos upon *RNaseH1* and *RNaseH1^{CD}* overexpression. RNase H treatment verifies specificity of antibody, and antibody specific for double-stranded DNA is used as a loading control. Oregon R is a wildtype control. Quantification of signal for 2 (Oregon R) or 3 (RNaseH and RNaseH^{CD}) biological replicates is to the right. * < 0.05, one-way ANOVA with Tukey's multiple comparisons test.

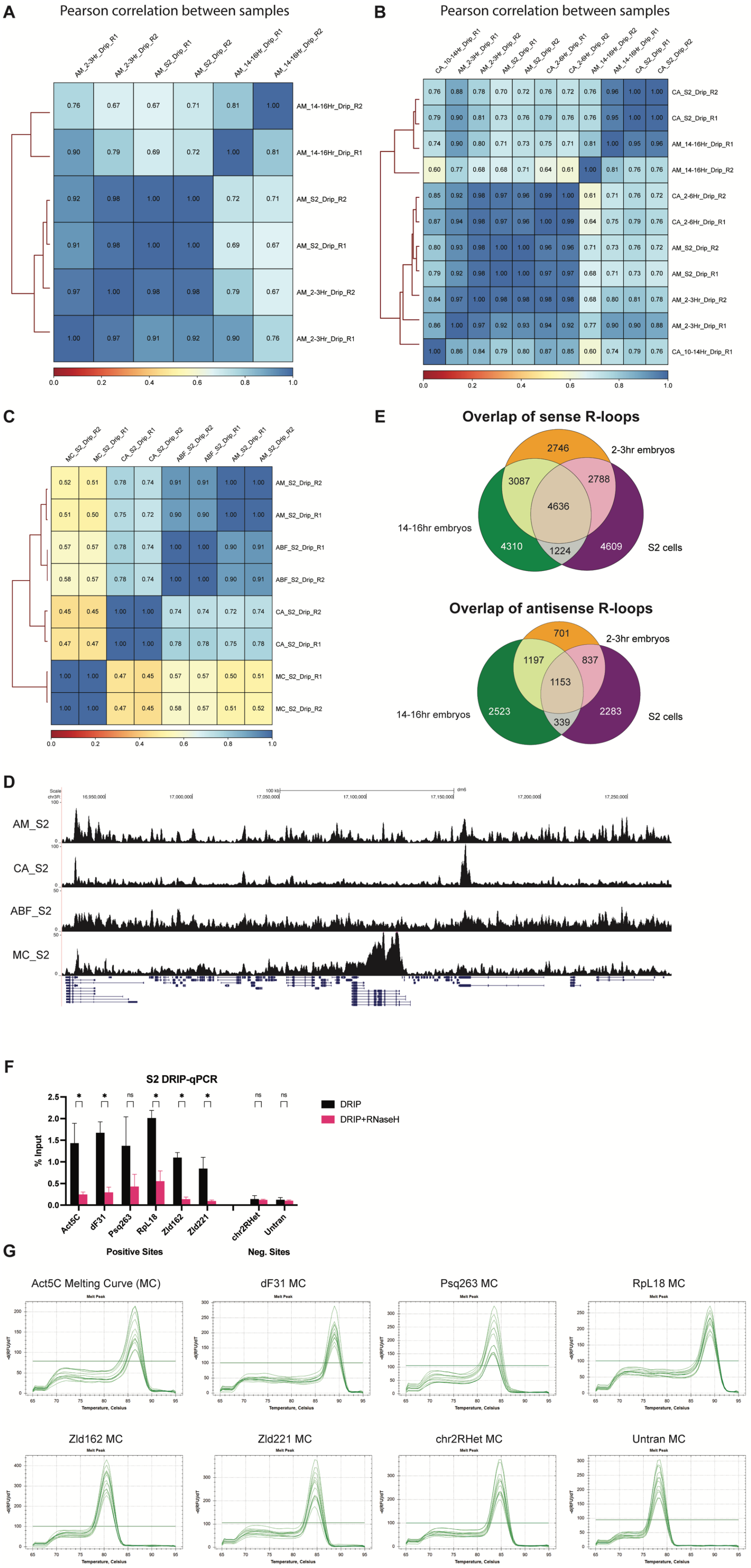
A
Pearson correlation between samples
AM_2-3Hr_Drip_R1
AM_2-3Hr_Drip_R2
AM_S2_Drip_R1
AM_S2_Drip_R2
AM_14-16Hr_Drip_R1
AM_14-16Hr_Drip_R2
B
Pearson correlation between samples
CA_10-14Hr_Drip_R1
CA_2-6Hr_Drip_R1
CA_2-6Hr_Drip_R2
AM_14-16Hr_Drip_R1
AM_14-16Hr_Drip_R2
CA_S2_Drip_R1
CA_S2_Drip_R2
C
MC_S2_Drip_R1
MC_S2_Drip_R2
ABF_S2_Drip_R1
ABF_S2_Drip_R2
E
Overlap of sense R-loops
2746
2-3hr embryos
3087
2788
4636
14-16hr embryos
4310
1224
4609
S2 cells
Overlap of antisense R-loops
701
2-3hr embryos
1197
837
1153
14-16hr embryos
2523
2283
339
S2 cells
D
AM_S2
CA_S2
ABF_S2
MC_S2
F
S2 DRIP-qPCR
DRIP
DRIP+RNaseH
% Input
Act5C
dF31
Psq263
RpL18
Zld162
Zld221
chr2RHet
Untran
Positive Sites
Neg. Sites
G
Act5C Melting Curve (MC)
dF31 MC
Psq263 MC
RpL18 MC
Zld162 MC
Zld221 MC
chr2RHet MC
Untran MC

Supplemental Figure 2: Properties of R-loops in Drosophila (A) Pearson's correlation between ssDRIP-seq replicates at 1kb resolution. AM- Alex Munden, this study. (B) Correlation between ssDRIP-seq replicates at 1kb resolution between this study (AM - Alex Munden) or data obtained from Alecki et al. 2020 (CA)[27] (C) Correlation at 1kb resolution between ssDRIP-seq replicates specifically from S2 cells. AM – this study; CA – Alecki et al. 2020[27]; ABF – Bayona-Feliu et al., 2017[74]; and MC – Crossley et al., 2020)[14] (D) Screen shot of unstranded ssDRIP-seq or DRIP-seq data for the same data sets as in C. (E) Venn diagrams of overlap of stranded R-loops between S2 cells, 2-3 hour embryos, and 14-16 hour embryos. Sense R-loops on top, antisense R-loops on bottom. (F) DRIP-qPCR validation of several R-loop positive and negative loci in S2 cells. (G) Melting curves for the qPCR products from the reactions in F.

A

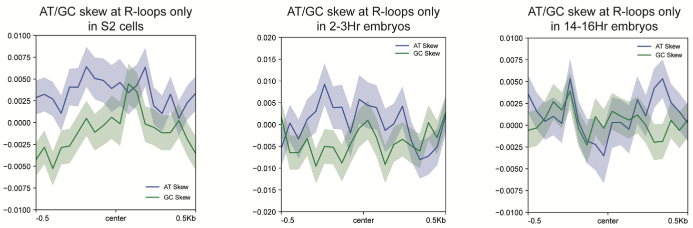

B

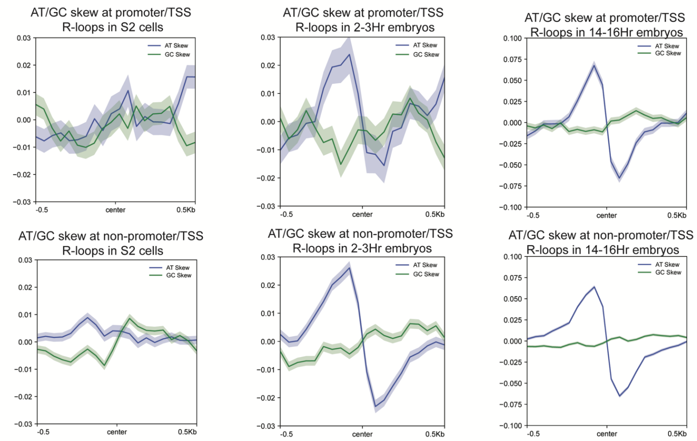

C

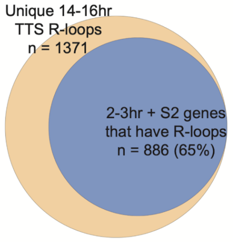

D

Rank	Motif	P-value	log P-pvalue	% of Targets	% of Background	STD(Bg STD)	Best Match/Details
1	AAAAAAAAAAAA	1e-1413	-3.254e+03	21.44%	8.73%	48.4bp (78.7bp)	hb/dmmpmm(Noyes)/fly(0.794) More Information | Similar Motifs Found
2	GGAGGAGGAGGA	1e-1352	-3.114e+03	31.82%	18.65%	51.4bp (70.0bp)	Trl/dmmpmm(Down)/fly(0.559) More Information | Similar Motifs Found
3	GGGGGGAG	1e-149	-3.432e+02	42.12%	36.14%	56.1bp (64.8bp)	Trl(Zf)/S2-GAGAfactor -ChIP-Seq(GSE40646)/Homer(0.647) More Information | Similar Motifs Found
4	CCCCTTCC	1e-127	-2.947e+02	27.92%	23.00%	55.2bp (68.4bp)	Kr/dmmpmm(Noyes)/fly(0.718) More Information | Similar Motifs Found
5	AACAACAACA	1e-106	-2.457e+02	14.88%	11.43%	56.1bp (66.6bp)	Aef1/dmmpmm(Pollard)/fly(0.864) More Information | Similar Motifs Found
6	GAGACAGA	1e-104	-2.398e+02	40.18%	35.22%	56.3bp (67.1bp)	Trl/MA0205.2/Jaspar(0.781) More Information | Similar Motifs Found
7	ATCATCATCA	1e-53	-1.239e+02	7.78%	5.96%	56.7bp (57.1bp)	ttk/MA0460.1/Jaspar(0.615) More Information | Similar Motifs Found
8	ACCACATAATGA	1e-52	-1.201e+02	0.13%	0.01%	58.2bp (42.2bp)	Btn/dmmpmm(Noyes_hd)/fly(0.690) More Information | Similar Motifs Found
9	GATTGGAGCTAA	1e-38	-8.804e+01	0.08%	0.00%	53.6bp (31.1bp)	POL013.1_MED-1/Jaspar(0.622) More Information | Similar Motifs Found
10	AAGTTCAGAAAT	1e-37	-8.600e+01	0.12%	0.01%	55.1bp (27.4bp)	ct/MA0218.1/Jaspar(0.627) More Information | Similar Motifs Found
11	GTAGTCCCAGGC	1e-36	-8.456e+01	0.07%	0.00%	51.8bp (18.8bp)	dl-B/dmmpmm(Bergman)/fly(0.559) More Information | Similar Motifs Found
12	AGGATGTTATGG	1e-36	-8.456e+01	0.07%	0.00%	53.5bp (38.4bp)	ara/dmmpmm(Noyes_hd)/fly(0.712) More Information | Similar Motifs Found
13	AAGTTGGGTGGC	1e-34	-7.985e+01	0.09%	0.01%	52.3bp (18.5bp)	Hr46/dmmpmm(Pollard)/fly(0.574) More Information | Similar Motifs Found
14	TGTGTCATGT	1e-34	-7.935e+01	2.89%	2.02%	56.0bp (60.3bp)	h/dmmpmm(Noyes)/fly(0.670) More Information | Similar Motifs Found
15	CAGTGAACCT	1e-33	-7.768e+01	0.07%	0.00%	56.8bp (15.9bp)	eyg/dmmpmm(Bergman)/fly(0.689) More Information | Similar Motifs Found
16	AGCGTTGCCTCG	1e-30	-7.018e+01	0.09%	0.01%	55.7bp (14.8bp)	POL010.1_DCE_S_III/Jaspar(0.589) More Information | Similar Motifs Found

Supplemental Figure 3: Sequence properties of R-loops in Drosophila (A) Metaplots of AT and GC skew at developmental-specific sites of R-loop formation (note the difference in scale for each window). Shaded regions represent the standard error of the mean. (B) Metaplots of AT and GC skew at promoter TSS sites and all other R-loops for each cell type. (C) Measurement of the R-loops found only at the TTS/3' UTR in the 14-16hr embryos and whether those genes have R-loops elsewhere in the gene in S2 and 2-3hr embryos. (D) The top 16 results from the HOMER motif analysis. Only P-values less than 1e-100 should be considered as potential motifs.

Chromatin associated factors differentially associate with R-loops

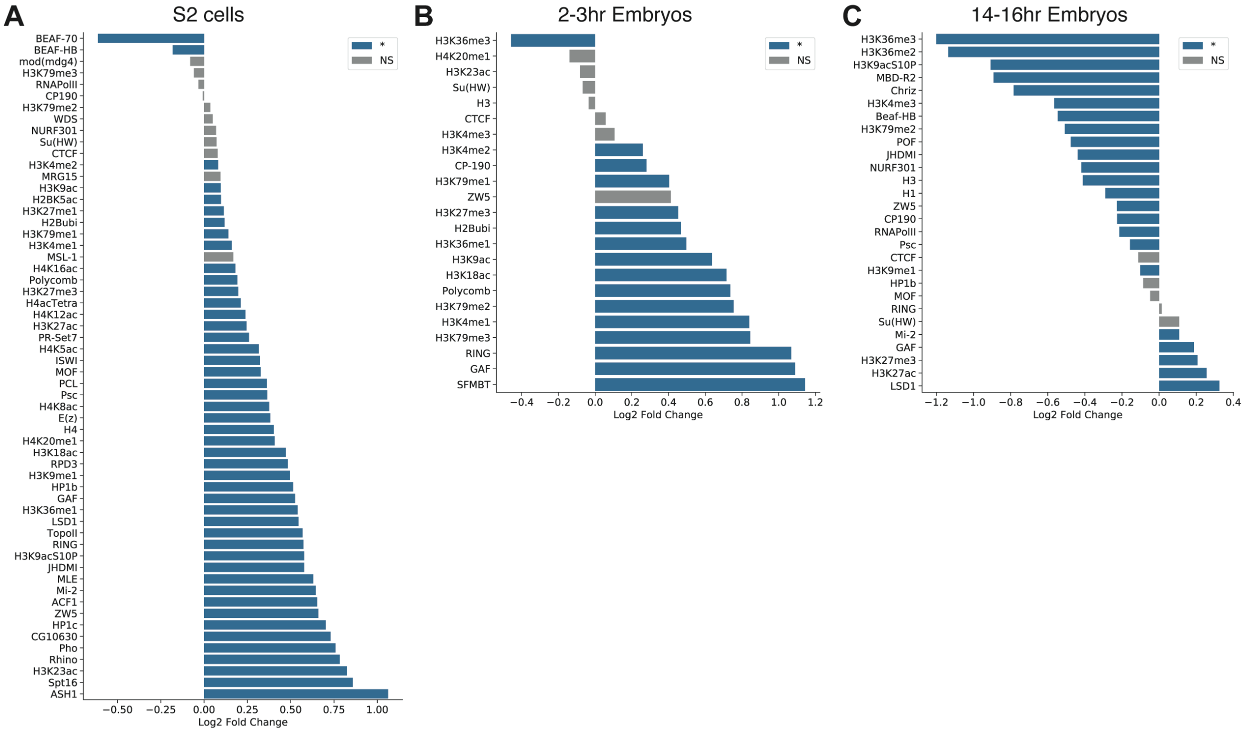

Supplemental Figure 4: Expanded chromatin associated factors associated with R-loops for every cell type. (A) Markers positively and negatively associated with R-loops in S2 cells. * < 0.05 with Bonferroni correction for multiple testing. (B) Same as A, except for 2-3 hour embryos. (C) Same as A, except for 14-16 hour embryos.
